# CsAGA1 and CsAGA2 Mediate RFO Hydrolysis in Partially Distinct Manner in Cucumber Fruits

**DOI:** 10.3390/ijms222413285

**Published:** 2021-12-10

**Authors:** Bing Hua, Mengying Zhang, Jinji Zhang, Haibo Dai, Zhiping Zhang, Minmin Miao

**Affiliations:** 1 College of Horticulture and Plant Protection, Yangzhou University, Yangzhou 225009, China; Binghua@yzu.edu.cn (B.H.); myzhang202112@163.com (M.Z.); MX120180598@yzu.edu.cn (J.Z.); DX120180088@yzu.edu.cn (H.D.); zhangzp@yzu.edu.cn (Z.Z.); 2 Joint International Research Laboratory of Agriculture and Agri-Product Safety of Ministry of Education of China, Yangzhou University, Yangzhou 225009, China; 3 Key Laboratory of Plant Functional Genomics of the Ministry of Education, Jiangsu Key Laboratory of Crop Genomics and Molecular Breeding, Yangzhou University, Yangzhou 225009, China

**Keywords:** cucumber, raffinose family oligosaccharides, sugar, α-galactosidase, fruit

## Abstract

A Raffinose family oligosaccharides (RFOs) is one of the major translocated sugars in the vascular bundle of cucumber, but little RFOs can be detected in fruits. Alpha-galactosidases (α-Gals) catalyze the first catabolism step of RFOs. Six α-Gal genes exist in a cucumber genome, but their spatial functions in fruits remain unclear. Here, we found that RFOs were highly accumulated in vascular tissues. In phloem sap, the stachyose and raffinose content was gradually decreased, whereas the content of sucrose, glucose and fructose was increased from pedicel to fruit top. Three alkaline forms instead of acid forms of *α-Gals* were preferentially expressed in fruit vascular tissues and alkaline forms have stronger RFO-hydrolysing activity than acid forms. By inducible gene silencing of three alkaline forms of *α-Gals*, stachyose was highly accumulated in RNAi-*CsAGA2* plants, while raffinose and stachyose were highly accumulated in RNAi-*CsAGA1* plants. The content of sucrose, glucose and fructose was decreased in both RNAi-*CsAGA1* and RNAi-*CsAGA2* plants after β-estradiol treatment. In addition, the fresh- and dry-weight of fruits were significantly decreased in RNAi-*CsAGA1* and RNAi-*CsAGA2* plants. In cucurbitaceous plants, the non-sweet motif within the promoter of *ClAGA2* is widely distributed in the promoter of its homologous genes. Taken together, we found RFOs hydrolysis occurred in the vascular tissues of fruits. *CsAGA1* and *CsAGA2* played key but partly distinct roles in the hydrolysis of RFOs.

## 1. Introduction

Cucumber (*Cucumis sativus* L.) is an important vegetable worldwide with great economic and nutritional value [1,2]. Unlike most plants, in which sucrose (Suc) is the predominant sugar transported in the vascular bundle, raffinose (Raf) family oligosaccharides (RFOs) are the major sugars transported between “source” organs and “sink” organs in cucumber [3,4,5,6,7]. Fruit is one of the most important sink organs in cucumber plants. However, in this organ, few RFOs are detected, and hexoses are the predominant free carbohydrates, indicating quick hydrolysis of imported RFOs in cucumber fruits [8,9]. RFOs consisting of stachyose (Sta) and Raf are translocated into sieve elements and hydrolysed into sucrose (Suc) in sink tissues after long-distance translocation [10,11]. There are two opinions about the location of RFOs hydrolyzation in fruits. Some researchers believe that the catabolism of Sta and Raf occurs in the pedicel. Evidence supporting this view include little RFOs, which were detected in fruits [3,12,13]. While the activity of enzymes catalysing RFOs catabolism, such as α-galactosidase, Suc synthase (SS, synthetic activity), SPS and UDP- galactose pyrophosphorylase were detected in the pedicel [6,14]. However, other researchers have suggested that RFOs are transported into the fruit and metabolized rapidly there. The evidence supporting this view include that RFOs exist in the cucurbits fruit [8,10,15,16]; exudates from the fruit’s main bundles contains Sta as the major sugar [12,17]; and active RFO metabolic enzymes, including alkaline a-galactosidase have been detected in the cucurbit’s fruits [10,11,12,15,18,19].

α-Galactosidase (EC3.2.1.22), also known as α-d-galactoside galactohydrolase and melibiase, is an exoglycosidase that hydrolyses the terminal nonreducing α-galactosyl moieties from glycolipids and glycoproteins [20,21]. α-galactosidases, distributed in various plant organs including seeds, seedlings, leaves and fruits, are involved in many physiological, biochemical processes, such as RFO unloading in fruits, seed development and germination, leaf development and senescence [21,22,23,24]. When RFOs were unloaded in cucumber fruits, previous studies showed that α-galactosidase catalysed the initial step of RFOs decomposition [8,18]. Taken together, α-galactosidase is a key determinant of fruit sink strength and an important regulator of source-sink balance in cucumber plants.

In higher plants, α-galactosidases are classified into two groups, acid α-galactosidases (GAL) and alkaline α-galactosidases (AGA), according to their activity in response to pH. There is a negative correlation between Sta levels and the activity of CsAGAs in cucumber peduncles, suggesting that CsAGAs rather than CsGALs may be responsible for the metabolism of imported Sta [12]. Irving et al. (1997) also reported that the activity of AGA was higher than that of GAL in *Cucurbita maxima* Duch. fruits at anthesis. Gao and Schaffer (1999) identified two AGAs in melon (*Cucumis melo* L.). CmAGA1 (AAM75139.1) showed significant activity with both Raf and Sta, whereas CmAGA2 (AAM75140.1) was relatively specific for Sta [19]. In addition, CmAGA1 enzyme activity is increased during the early stages of melon ovary development and fruit set, while CmAGA2 enzyme activity is declined during this period, indicating CmAGA1 may play a key role in melon photoassimilate unloading [18,19]. However, ClAGA2, an alkaline α-galactosidase, was recently identified as the key factor controlling the hydrolysis of Sta and Raf in watermelon (*Citrullus lanatus*); *claga2* mutants showed reduced content of Glu, Fru and Suc, but increased Raf content in the fruit fresh of watermelon [5,6]. In addition, two single-nucleotide polymorphisms (SNPs) existing in the *ClAGA2* promoter affected the binding of the transcription factor ClNF-YC2 to regulate *ClAGA2* expression [5]. In cucumber, there are six putative α-galactosidase genes (*α-Gals*), with three acid forms (*CsGAL1*, *CsGAL2*, *CsGAL3*) and three alkaline forms (*CsAGA1*, *CsAGA2*, *CsAGA3*) [25]. Those six α-Gals are universally expressed in different organs and have different substrate specificities, pH and temperature responding curves in vitro [25]. However, little is known about which forms is responsible for RFOs hydrolysis when they are unloaded into cucumber fruits.

The vascular systems, consisting of xylem and phloem, play key roles in the translocation of solutes [9,26,27]. Xylem and phloem play partly different roles: xylem mainly transports water and solute minerals, and phloem mainly transports photosynthetic products from source to sink organs [26,27]. Different from many other plants, phloem tissues in cucumber include two systems: fascicular phloem and extrafascicular phloem [28,29,30]. For unloading RFOs in fruits, previous work indicated that Sta may be quickly broken down to Suc by a-Gals and subsequently hydrolysed to hexose after reaching the vascular bundles of fruits [3,9]. In addition, there are higher Raf content and lower Sta content in stalk than pedicel [9]. The distinct distribution of Sta and Raf indicated the hydrolysis of Sta may differ from Raf hydrolysis.

Although a series of studies showed that a-Gals play important roles in RFO hydrolyzation, the location of RFO hydrolysis and the type of functional a-Gals are not fully resolved. Thus, our major objectives were (1) to explore where RFOs were hydrolysed in the fruit; (2) to confirm which α-galactosidases participated in the RFOs hydrolyzation in the fruit; (3) to verify how RFOs were hydrolysed by α-galactosidases in the fruits.

## 2. Results

### 2.1. The Distribution Pattern of Soluble Sugars

**Figure 1:**
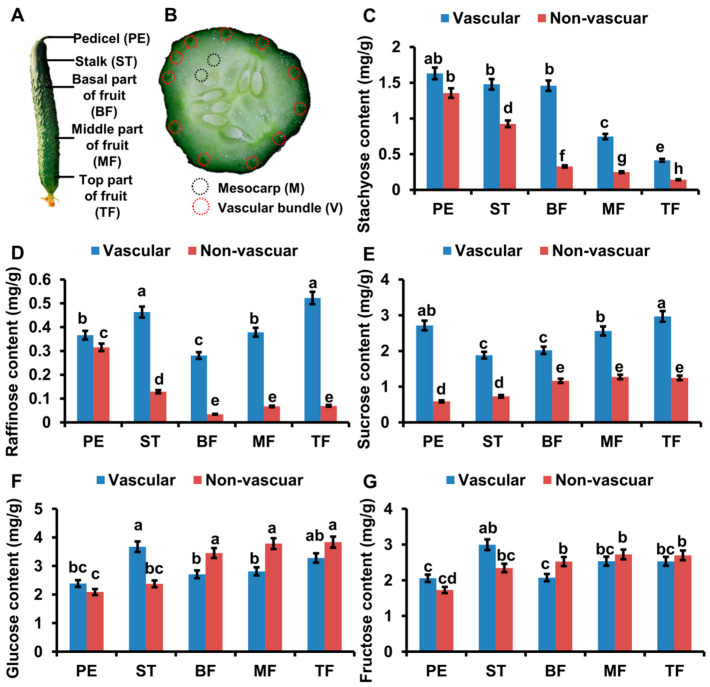
High accumulation of soluble sugar in vascular tissue of fruits. (**A**,**B**) The schematic diagram shows the sampling location in fruit. Vascular tissue and non-vascular tissue of pedicel (PE), stalk (ST), basal part (BF), middle part (MF) and top part (TF) of fruit were used for the analysis of soluble sugar content. The red circles show the vascular bundle; the white circles show the mesocarp. (**C**–**G**) The stachyose (Sta) (**C**), raffinose (Raf) (**D**), sucrose (Suc) (**E**), glucose (Glu) (**F**) and fructose (Fru) (**G**) content in different tissue of fruit were analysed by HPLC. The bars represent standard deviation (SD) of five biological replicates. Bars marked with different letters denote significant differences (*p* < 0.05) from Student’s *t*-test.

To investigate the location of RFOs hydrolysis, we examined the distribution of soluble sugar (Sta, Raf, Suc, Glu and Fru) in cucumber fruits and selected the vascular and non-vascular tissues of pedicel (PE), stalk (ST), basal part (BF), middle part (MF) and top part (TF) of fruits for further analysis (Figure 1A,B).

The content of Sta in non-vascular tissues was highest in pedicel and gradually decreased from BF to TF; while in vascular tissues, Sta content was higher in PE, ST, and BF (Figure 1C). In non-vascular tissues, the Raf content was gradually decreased from PE to TF; while in vascular tissues, Raf content was high in ST and TF, but low in PE, BF and MF (Figure 1D). The content of Suc, Glu and Fru was gradually increased from PE to TF in both vascular and non-vascular tissues (Figure 1E–G). Taken together, RFOs decreased from PE to TF while the content of Suc, Glu and Fru was increased from PE to TF (Figure 1C–G). In addition, RFO content was significantly higher in vascular tissues than non-vascular tissues (Figure 1C–G). To verify whether the distribution of soluble sugars is conserved in *Cucumis* plants, we selected a fresh-eating cucumber cultivar “83-16” (North European greenhouse ecotype), a Vietnamese melon (named melon below), and a non-sweet melon cultivar, for further analysis. The RFOs content was gradually decreased from PE to TF, while Glu, Fru contents were gradually increased from PE to TF in vascular tissues of “83-16” and melon (Supplemental Figures S1A–G and S3A–G). In addition, RFOs content in vascular tissues of melon fruits was significantly higher than in mesocarp, which is consistent with the trend in cucumber (Figure 1D, Figures S1C and S3C). Those results indicated that RFOs hydrolysis could occur mainly in vascular tissues and is conserved in *Cucumis* plants including cucumber.

### 2.2. The Distribution of Soluble Sugars in Phloem Sap

**Figure 2:**
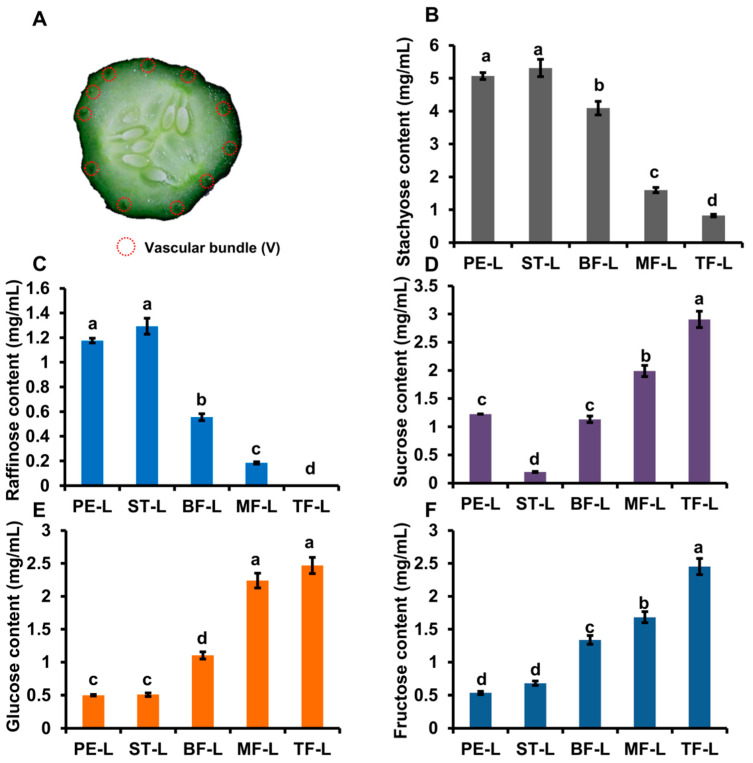
The distribution of soluble sugar in phloem sap of fruits. (**A**) The schematic diagram shows the collected sites of phloem sap. (**B**–**F**) The Sta (**B**), Raf (**C**), Suc (**D**), Glu (**E**) and Fru (**F**) content of phloem sap in pedicel (PE), stalk (ST), basal part(BF), middle part(MF) and top part(TF) of fruit. The bars represent standard deviation (SD) of five biological replicates. Bars marked with different letters denote significant differences (*p* < 0.05) from Student’s *t*-test.

To further verify whether vascular tissues are the main location of RFOs hydrolysis, we selected the fascicular phloem sap of PE, ST, BF, MF and TF for soluble sugar measurement (Figure 2A).

Sta content of phloem sap was highest and up to 5 mg/mL in PE and ST; Sta content was gradually reduced from BF to TF (Figure 2B). Similar with Sta, Raf content of phloem sap was highest in PE, ST and gradually reduced from BF to TF (Figure 2C). It was odd that Suc content was lowest in ST and only about 0.2 mg/mL (Figure 2D). In contrast with Sta and Raf, the content of Suc, Glu and Fru in phloem sap was low in PE, ST and gradually increased from BF to TF (Figure 2D–F).

We also analysed soluble sugar content in “83-16” and melon. In “83-16”, Sta and Raf contents were highest in S, PE and reduced from ST to TF; the content of Glu and Fru was low in S, PE and gradually increased from ST to TF (Supplemental Figure S1H–L). In melon, Sta content was high in PE, ST and was sharply reduced in MF and TF; Raf content was gradually decreased from PE to TF (Supplemental Figure S3H,I). Suc content was low in PE, ST and was high in MF, TF; Glu content was similar in PE, ST, MF and TF; Fru content was lowest in PE (Supplemental Figure S3J–L). Those results showed that RFO hydrolysis in phloem sap mainly occurred in the pedicel and stalk and displayed a similar trend in cucumber and melon.

### 2.3. The Expression Pattern of α-Gals in Fruits

**Figure 3:**
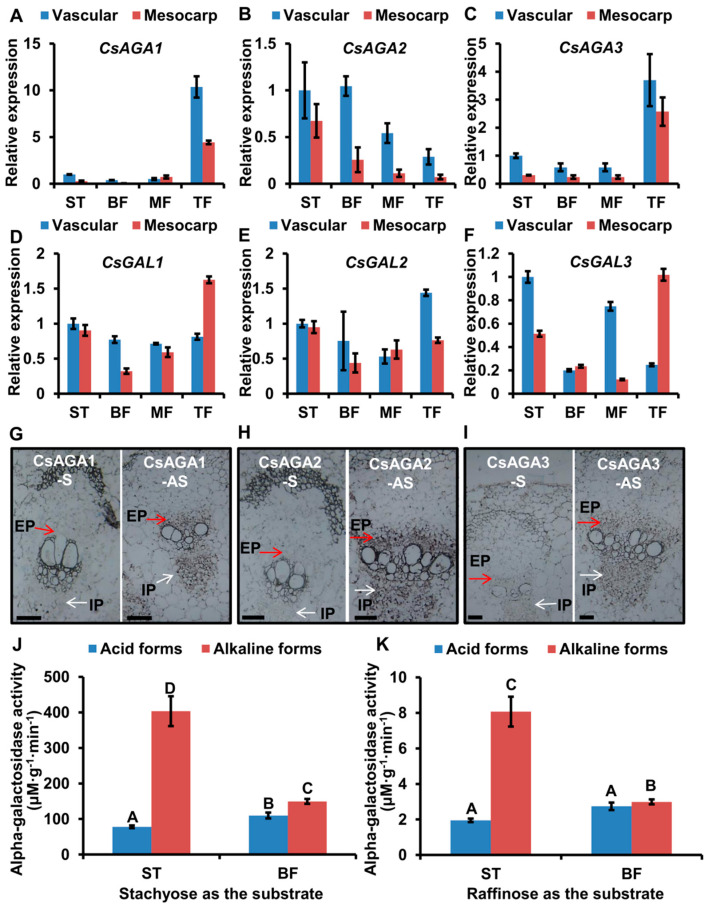
The expression of *α-galactosidase* genes in fruit. (**A**–**C**) Relative expression showed that *α-galactosidase* genes of alkaline forms (*CsAGA1* (**A**), *CsAGA2* (**B**) and *CsAGA1* (**C**)) were preferred expressed in phloem tissue. (**D**,**E**) Relative expression showed that *α-galactosidase* genes of acid forms (*CsGAL1* (**D**), *CsGAL2* (**E**) and *CsGAL3* (**F**)) did not show the prefer expression in phloem tissue by qRT-PCR. The bars represent standard deviation (SD) of three biological replicates. *18S rRNA* acted as reference gene and relative amounts were normalized with respect to the expression in vascular of stalk. (**G**–**I**) In situ hybridizations showed that *α-galactosidase* genes of alkaline forms (*CsAGA1* (**G**), *CsAGA2* (**H**) and *CsAGA3* (**I**)) were prefer expressed in phloem. The anti-sense probe was used for detecting the expression of *α-galactosidase* genes of alkaline forms (right). The sense probe was used for in situ hybridization in fruit as a negative control (left). Vascular bundles were labelled, with the IP (internal phloem) marked with white arrows and EP (external phloem) marked with red arrows. Bars = 100 μm. (**J**,**K**) Alpha-galactosidase activity of alkaline forms was higher than acid forms when Sta (**J**) or Raf (**K**) acted as the substrate. Alpha-galactosidase activities in stalk (ST) and basal part (BF) of fruit were measured.

In watermelon, a previous study showed that *α-Gals* play key roles in RFO hydrolysis and are highly expressed in vascular bundles [5]. So, the high expression of *α-Gals* in vascular tissues probably contributes to the hydrolysis of RFOs in cucumber. Our previous study showed that there are six α-Gals, three acid forms (*CsGAL1*, *CsGAL2* and *CsGAL3*) and three alkaline forms (*CsAGA1*, *CsAGA2*, *CsAGA3*) in cucumber [25]. To verify which α-Gals is involved in RFO hydrolysis in cucumber, we analysed their expression in fruits. We selected the vascular tissues and non-vascular tissues of ST, BF, MF and TF for analysis. By qRT-PCR, we found that *CsAGAs* were highly expressed in vascular tissues: *CsAGA1* and *CsAGA3* were highly expressed in TF; the expression of *CsAGA2* was gradually decreased from ST to TF (Figure 3A–C). Different from *CsAGAs*, *CsGALs* did not show the preferred expression in vascular tissues (Figure 3D–F). Notably *CsAGA2* expression displayed a similar trend with Sta distribution in fruits. To visualize the spatial expression pattern of alkaline forms α-Gals (*CsGAL1*, *CsGAL2*, *CsGAL3*) in vascular tissues, we conducted in situ hybridizations. As shown in Figure 3G–I, there were clear hybridization signals in EP (external phloem) and IP (internal phloem), while no obvious signal in mesocarp cells using the anti-sense probe. Among them, in situ hybridization signal of *CsAGA2* was stronger than *CsAGA*1 and *CsAGA3* (Figure 3G–I). In contrast to the anti-sense probe, there were no obvious signals, either in phloem or in mesocarp (Figure 3G–I). We further compared the α-galactosidase activity of acid forms and alkaline forms in ST and BF. In ST and BF, α-Gals activity of alkaline forms was higher than acid forms when Sta or Raf acted as the substrate (Figure 3J,K). Those combined results implied three alkaline forms α-Gals, but not acid forms α-Gals, might be involved in RFOs hydrolysis in vascular bundles.

With the similar soluble sugar distribution in the fruit of cucumber, “83-16” and melon, the expression pattern of *α-Gals* may also be similar. To verify it, we characterized the expression of *α-Gals* in “83-16” and melon by qRT-PCR. In “83-16”, *CsAGA2* was highly expressed in vascular tissues, but other five *α-Gals* did not show the vascular preferred expression (Supplemental Figure S2A–F). Similar with cucumber, three alkaline forms *α-Gals* were highly expressed in vascular tissues, while acid forms *α-Gals* did not display the vascular preferred expression in melon (Supplemental Figure S4A–F). The high expression level of alkaline forms *α-Gals,* especially *CsAGA2* in vascular tissues implies their functional involvement in RFOs hydrolysis, so we chose CsAGA2 for further functional verification.

### 2.4. CsAGA2 Mediates Stachyose Hydrolysis

Our previous works showed that *CsAGAs* are universally expressed in different cucumber organs, and *α-Gal* genes may regulate other physiological processes including RFOs hydrolyzation [25]. To avoid potential artifacts caused by constitutive silencing, we silenced *CsAGAs* expression using an β-estradiol inducible expression system (Supplemental Figure S5A,B). As the content of RFOs is high in pedicel, we treated the pedicel with 150 μM β-estradiol once a day.

**Figure 4:**
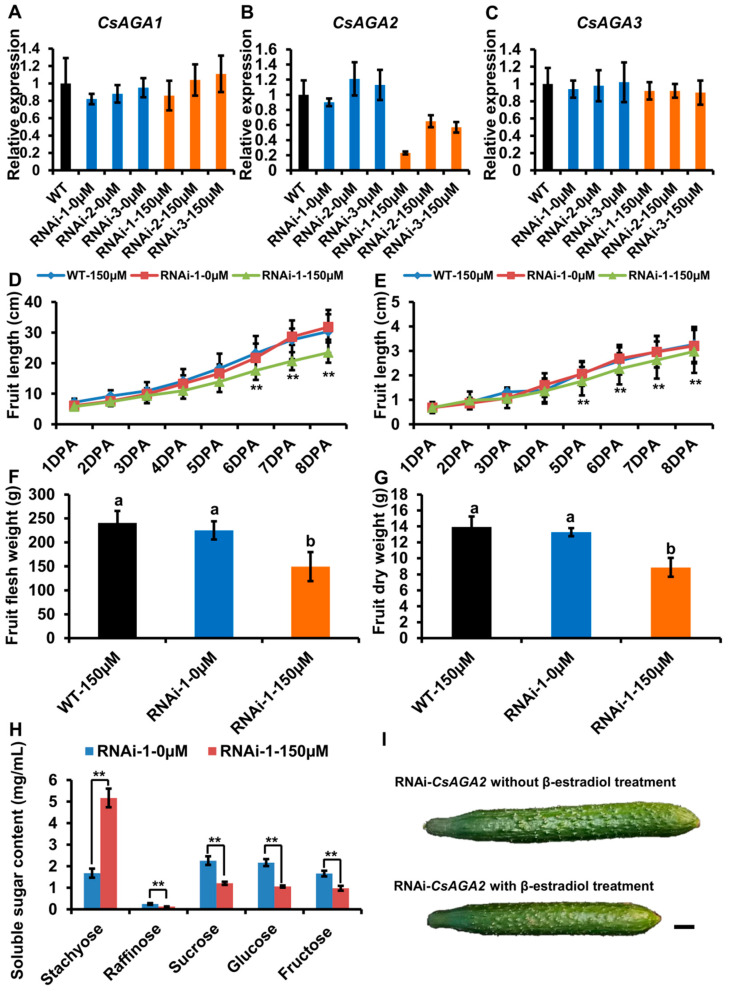
*CsAGA2* mainly regulated stachyose hydrolysis but not Raf. (**A**–**C**) qRT-PCR analysis of the expression of *CsAGA1* (**A**), *CsAGA2* (**B**) and *CsAGA3* (**C**) in RNAi-*CsAGA2* plants with or without β-estradiol treatment. The expression of *CsAGA2,* but not *CsAGA1* (**A**) and *CsAGA3* (**C**), was down regulated in RNAi-*CsAGA2* plants with 150 μM β-estradiol treatment but not regulated in RNAi-*CsAGA2* plants without β-estradiol treatment (**B**). Three lines of RNAi-*CsAGA2* were chosen for qRT-PCR analysis. The bars represent standard deviation (SD) of three biological replicates. *18S rRNA* act as reference gene and relative amounts were normalized with respect to the expression in WT. (**D**,**E,I**) The fruit length (**D,I**) and fruit diameter (**E,I**) were decreased in RNAi-*CsAGA2* plants treated with β-estradiol compared with WT with β-estradiol treatment and RNAi-*CsAGA2* plants without β-estradiol treatment. The fruits at 1DPA (day post-anthesis), 2DPA, 3DPA, 4DPA, 5DPA, 6DPA, 7DPA and 8DPA stages were selected for measurement. The bars represent standard deviation (SD) of fifteen biological replicates. (**F**,**G**) The fruit fresh weight (**F**) and dry weight (**G**) were reduced in RNAi-*CsAGA2* plants treated with β-estradiol compared with WT with β-estradiol treatment and RNAi-*CsAGA2* plants without β-estradiol treatment. The fruit at 7DPA stage were selected for measurement. The bars represent standard deviation (SD) of 15 biological replicates. Bars marked with different letters denote significant differences (*p* < 0.05) from Student’s t-test. (**H**) The analysis of soluble sugar content in RNAi-*CsAGA2* plants with or without β-estradiol treatment. Soluble sugar of phloem sap was collected for measurement. Sta, Raf, Suc, Glu and Fru were measured. The bars represent standard deviation (SD) of fifteen biological replicates. Bars annotated with asterisks are significantly different according to Fisher’s least significant difference test after ANOVA (**, *p* < 0.01). (**I**) The fruit phenotype comparison of RNAi-*CsAGA2* plants with or without β-estradiol treatment. Bar = 1 cm.

After β-estradiol treatment, we characterized *CsAGA2* expression by qRT-PCR. The result showed that *CsAGA2* expression was significantly down-regulated in the pedicel of RNAi-*CsAGA2* plants after β-estradiol treatment (Figure 4B). To exclude the potential nonspecific inhibition of *CsAGA1* and *CsAGA3* expression, we also examined the expression of both *CsAGA1* and *CsAGA3*, and found no significant difference in RNAi-*CsAGA2* plants with β-estradiol treatment compared with wild type or RNAi-*CsAGA2* plants without β-estradiol treatment (Figure 4A,C). Those results showed that β-estradiol treatment could specifically silence the expression of *CsAGA2* in RNAi-*CsAGA2* plants.

After β-estradiol treatment, fruit development was obviously blocked in RNAi-*CsAGA2* plants (Figure 4I). To evaluate the fruit morphology in RNAi-*CsAGA2*, we quantified the fruit length and diameter from 1DPA (day post-anthesis) stage to 8DPA stage. The fruit length was significantly reduced, while fruit diameter had no significant change in RNAi-*CsAGA2* plants with β-estradiol treatment compared with wild type treated with β-estradiol, or RNAi-*CsAGA2* plants without β-estradiol treatment (Figure 4D,E). Furthermore, we characterized the fresh weight and dry weight of fruits. At 8DPA stage, the fresh weight and dry weight of fruits were significantly decreased in RNAi-*CsAGA2* plants with β-estradiol treatment compared with wild type treated with β-estradiol or RNAi-*CsAGA2* plants without 0 μM β-estradiol treatment (Figure 4F,G).

As *CsAGA2* is highly expressed in vascular tissues where RFO hydrolysis mainly occurs, we characterized the soluble sugar contents of phloem sap. As expected, Sta content was higher, while Suc, Glu and Fru contents were lower in RNAi-*CsAGA2* plants treated with β-estradiol than without β-estradiol treatment (Figure 4H). Interestingly, Raf content was decreased in RNAi-*CsAGA2* after β-estradiol treatment (Figure 4H). Besides, the non-sweet motif (CTTAGGTTGGTGTTAGTG) also existed in the *CsAGA2* promoter (Supplemental Figure S6) [5]. Those results indicated that *CsAGA2* was the main gene contributing to Sta hydrolysis, and the other two alkaline forms α-Gals may play key roles in Raf hydrolysis.

### 2.5. CsAGA1 Mediates Both Stachyose and Raffinose Hydrolysis

**Figure 5:**
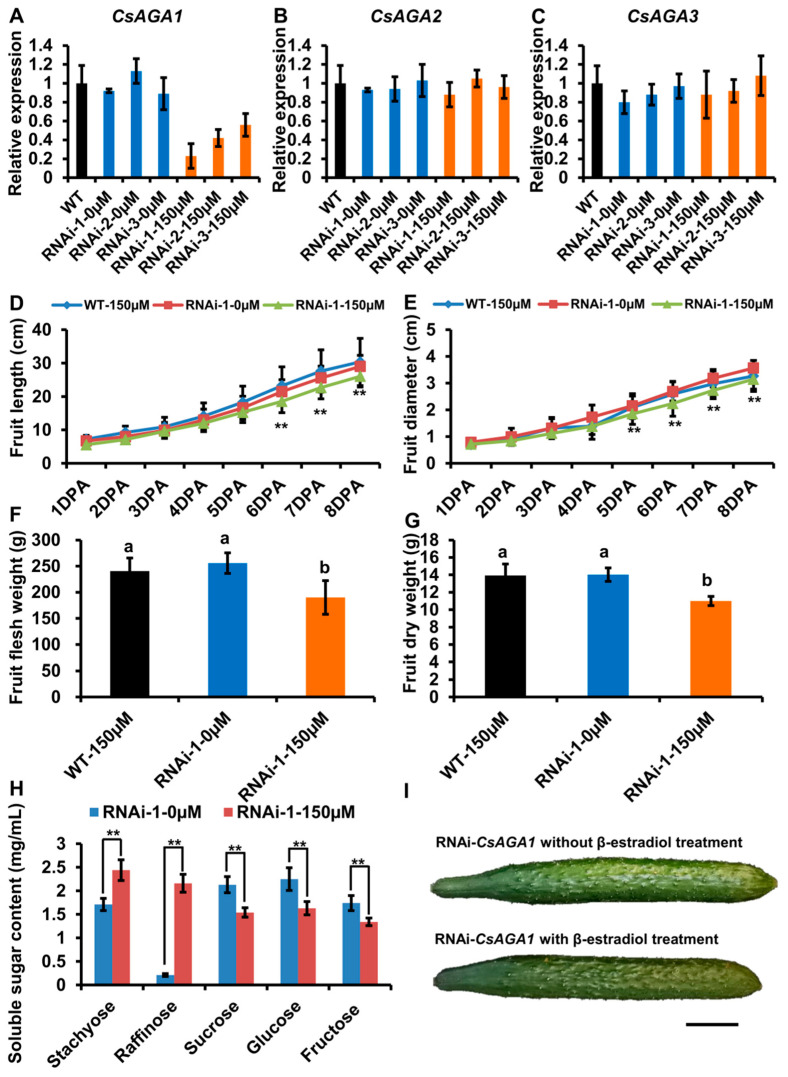
*CsAGA1* mainly regulates Raf hydrolysis but not Sta. (**A**–**C**) qRT-PCR analysis of the expression of *CsAGA1* (**A**), *CsAGA2* (**B**) and *CsAGA3* (**C**) gene in RNAi (RNA interfere)-*CsAGA1* plants with or without β-estradiol treatment. The expression of *CsAGA1* was down regulated in RNAi-*CsAGA1* plants with β-estradiol treatment, but not in RNAi-*CsAGA1* plants without β-estradiol treatment (**A**). The expression of *CsAGA2* (**B**) and *CsAGA3* (**C**) were not regulated in RNAi-*CsAGA1* plants after β-estradiol treatment. Three lines of RNAi-*CsAGA2* were chosen for qRT-PCR analysis. The bars represent standard deviation (SD) of three biological replicates. *18S rRNA* act as reference gene and relative amounts were normalized with respect to the expression in WT. (**D**,**E,I**) The fruit length (**D,I**) and fruit diameter (**E,I**) were decreased in RNAi-*CsAGA1* plants treated with β-estradiol compared with WT treated 150 μM β-estradiol, RNAi-*CsAGA1* plants without β-estradiol treatment. The fruit at 1DPA (day post anthesis), 2DPA, 3DPA, 4DPA, 5DPA, 6DPA, 7DPA and 8DPA stages were selected for measurement. The bars represent standard deviation (SD) of fifteen biological replicates. (**F**,**G**) The fruit fresh weight (**F**) and dry weight (**G**) were reduced in RNAi-*CsAGA1* plants treated with β-estradiol compared with WT treated with β-estradiol, RNAi-*CsAGA1* plants without β-estradiol treatment. The fruits at 7DPA stage were selected for measurement. The bars represent standard deviation (SD) of fifteen biological replicates. Bars marked with different letters denote significant differences (*p* < 0.05) from Student’s *t*-test. (**H**) The analysis of soluble sugar contents in RNAi-*CsAGA1* plants with or without β-estradiol treatment. Soluble sugar contents of phloem sap were collected for measurement. Sta, Raf, Suc, Glu and Fru contents were measured. The bars represent standard deviation (SD) of fifteen biological replicates. Bars annotated with asterisks are significantly different according to Fisher’s least significant difference test after ANOVA (**, *p* < 0.01). (**G**) The fruit phenotype comparison of RNAi-*CsAGA1* plants with or without β-estradiol treatment. Bar = 1 cm.

To verify the role of *CsAGA1* and *CsAGA3* in Raf hydrolysis, we generated the RNAi-*CsAGA1* and RNAi-*CsAGA3* plants using the similar strategy as RNAi-*CsAGA2*. In RNAi-*CsAGA1* plants, the expression of *CsAGA1*, but not *CsAGA2* and *CsAGA3*, was specifically suppressed after β-estradiol treatment (Figure 5A–C). Similarly, the fruit development was obviously blocked in RNAi-*CsAGA1* plants after β-estradiol treatment (Figure 5I). Furthermore, we quantified the fruit length and diameter. The fruit length was decreased and fruit diameter had no significant change in RNAi-*CsAGA1* plants after β-estradiol treatment (Figure 5D,E). The fruit fresh weight and dry weight were reduced in RNAi-*CsAGA1* plants after β-estradiol treatment (Figure 5F,G). To characterize the effect of *CsAGA1* in RFOs hydrolysis, we compared the soluble sugar content in RNAi-*CsAGA1* plants treated with or without β-estradiol. Sta and Raf contents were higher in RNAi-*CsAGA1* plants treated with β-estradiol than in those without β-estradiol (Figure 5H). Suc, Glu and Fru contents were reduced in RNAi-*CsAGA1* plants after β-estradiol treatment (Figure 5H). Those results indicated that *CsAGA1* played an essential role in RFOs (especially Raf) hydrolysis.

**Figure 6:**
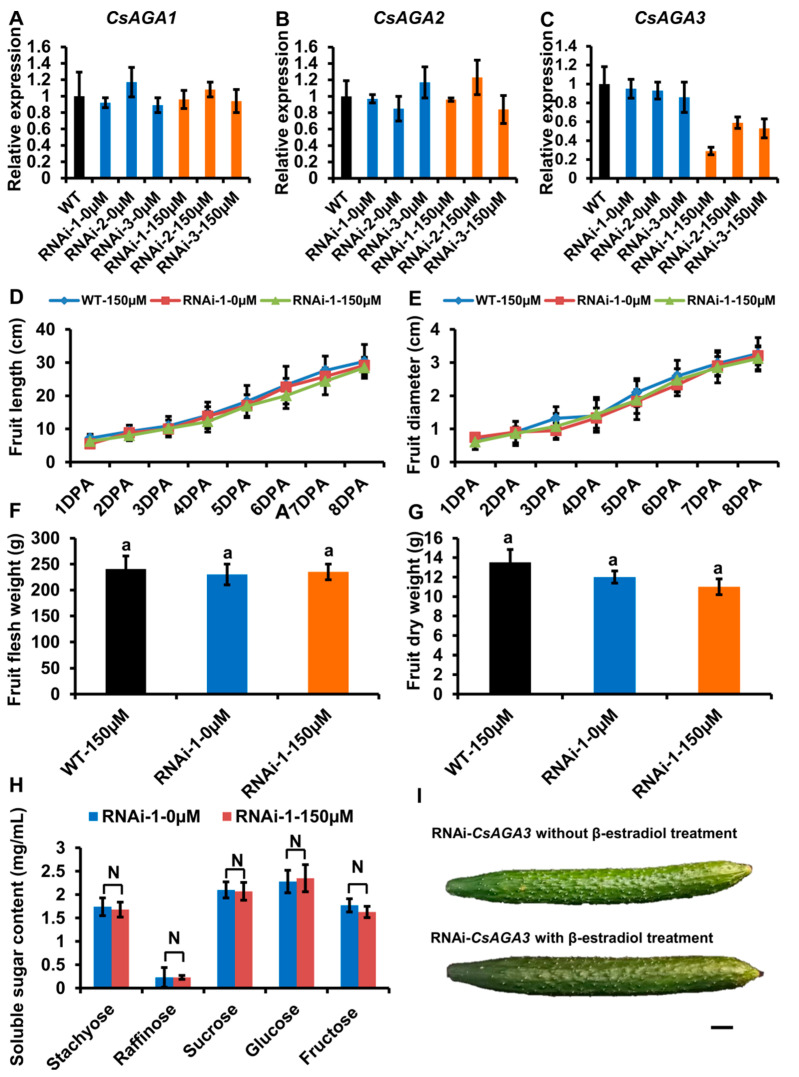
CsAGA3 did not regulated stachyose and raffinose hydrolysis. (**A-C**) qRT-PCR analysis of the expression of *CsAGA1* (**A**), *CsAGA2* (**B**) and *CsAGA3* (**C**) in RNAi-*CsAGA3* plants with or without β-estradiol treatment. The expression of *CsAGA3* was down regulated in RNAi-*CsAGA3* plants treated with β-estradiol, but not in RNAi-*CsAGA3* plants without β-estradiol treatment (**B**). The expression of *CsAGA1* (**A**) and *CsAGA2* (**B**) were not regulated in RNAi-*CsAGA3* plants treated with 150μM β-estradiol. Three lines of RNAi-*CsAGA2* were chosen for qRT-PCR analysis. The bars represent standard deviation (SD) of three biological replicates. *18S rRNA* acted as reference gene and relative amounts were normalized with respect to the expression in WT. (**D**,**E**,**I**) The fruit length (**D**,**I**) and fruit diameter (**E**,**I**) had no significant difference in RNAi-*CsAGA3* plants with β-estradiol treatment compared with WT with β-estradiol treatment, RNAi-*CsAGA3* plants without β-estradiol treatment. The fruit at 1DPA (day post anthesis), 2DPA, 3DPA, 4DPA, 5DPA, 6DPA, 7DPA and 8DPA stages were selected for measurement. The bars represent standard deviation (SD) of fifteen biological replicates. (**F**,**G**) The fruit flesh (**F**) weight and dry weight (**G**) had no significant change in RNAi-*CsAGA3* plants treated with β-estradiol compared with WT treated with β-estradiol, RNAi-*CsAGA3* plants without β-estradiol treatment. The fruit at 7DPA stage were selected for measurement. The bars represent standard deviation (SD) of fifteen biological replicates. Bars marked with different letters denote significant differences (*p* < 0.05) from Student’s t-test. (**H**) The analysis of soluble sugar contents in RNAi-*CsAGA3* plants with or without β-estradiol treatment. Soluble sugar contents of phloem sap were collected for measurement. Sta, Raf, Suc, Glu and Fru were measured. The bars represent standard deviation (SD) of fifteen biological replicates. Bars annotated with asterisks are significantly different according to Fisher’s least significant difference test after ANOVA (**, *p* < 0.01). (**G**) The fruit phenotype comparison of RNAi-*CsAGA3* plants with or without β-estradiol treatment. Bar = 1 cm.

Similarly, we characterized the phenotype of RNAi-*CsAGA3* plants. In RNAi-*CsAGA3* plants, the expression of *CsAGA3,* but not *CsAGA1* and *CsAGA2,* was suppressed after β-estradiol treatment (Figure 6A–C). The fruit length and diameter had no significant difference in RNAi-*CsAGA3* plants after β-estradiol treatment (Figure 5D,E). The fresh weight, dry weight and soluble sugar contents had no significant difference in RNAi-*CsAGA3* plants after β-estradiol treatment (Figure 5F–H).

## 3. Discussion

A series of works have shown that RFOs have a wide range of physiological functions in higher plants. As storage carbohydrates, RFOs are distributed in many tissues including seeds, leaves, fruits and so on [30,31,32]. As sugars being translocated, RFOs are found in the vascular bundle of Cucurbitaceae, Lamiaceae, Oleaceae, Scrophulariaceae and other several specials [21,30,31]. However, there are still a lot of gaps in answering how plants unload and hydrolyse RFOs.

### 3.1. A Chemical-Inducible Gene Silencing System in Pedicel of Cucumber Fruit

Transgenic technology is a powerful tool in basic plant biology research. Constitutive expression systems are usually used to express DNA fragment using constitutive promoters [32,33]. However, constitutive expression systems have several limits, such as pleiotropy, continuous expression of foreign genes, adverse effects on plant development and so on. Compared with constitutive expression systems, chemical-inducible systems offer numerous advantages including the expression at a given developmental stage for a specific duration [34,35]. Among them, XVE system is widely used in regulating transgene expression for its reliability, efficiency, and non-toxicity to plants [35,36,37,38]. In cucumber, α-Gals play a potential role in the unloading of sugar [25]. A-Gals are widely expressed in different organs, including seed, leaf, root and so on, and involved in leaf development, seed maturation and germination [9,23,25,39,40,41]. To study the function of α-Gals in unloading sugar, specifically in fruits, we treated RNAi-*CsAGA2* plants with β-estradiol of various concentrations in pedicel. RNAi-*CsAGA2* plants with 150 mM β-estradiol treatment displayed the lowest expression of *CsAGA2* (Supplemental Figure S5A,B). So, the XVE system is an efficient system to induce gene expression in cucumber pedicel and 150 mM is a proper concentration for gene induction.

### 3.2. Hydrolysis of RFOs in Fruits

**Figure 7:**
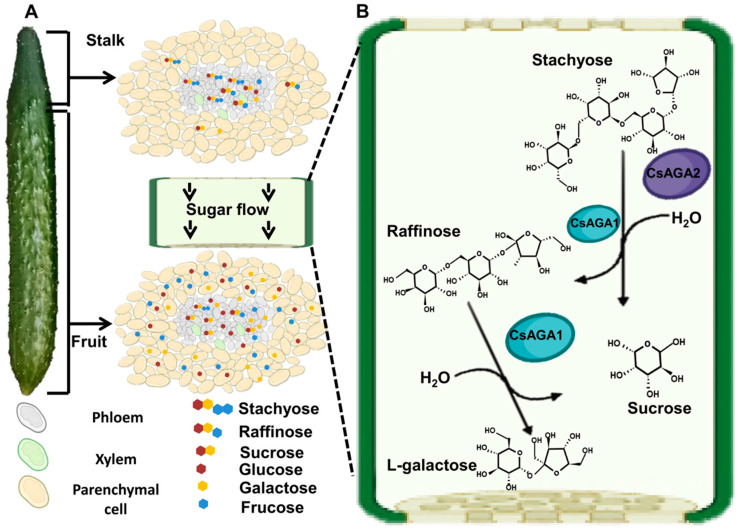
Proposed model of soluble sugar distribution and the process of Raf family oligosaccharides hydrolysis by CsAGA1, CsAGA2. (**A**) The soluble sugar distribution in fruit. Sta and Raf contents in phloem sap are gradually decreased from BF to TF. Suc, Glu and Fru contents are gradually increased in phloem sap from BF to TF. RFOs contents are higher in vascular tissues than in non-vascular tissues. (**B**) CsAGA1 hydrolyses Raf and Sta; CsAGA2 mainly controls Sta hydrolysis. Purple circle indicates CsAGA2, and green circle indicates CsAGA1.

Previous experiments indicated that pedicel has higher Sta content and lower Raf content, while stalk has relatively higher Raf content [8,9]. Those works suggest that Sta and Raf from leaves are quickly metabolized into monosaccharides (Glu, Fru) until they reach the stalk, and the regulation of Sta and Raf hydrolysis is different [8,9]. In this study, we measured the soluble sugar content and found that the RFO content was high in vascular tissues. Furthermore, we collected the phloem sap and measured soluble contents. In vascular tissues, non-vascular tissues, and phloem sap, Sta contents were highest in stalk and decreased from the stalk to fruit top (Figure 1C and Figure 7A). So, Sta might be mainly hydrolysed in the stalk part of fruits. The Sta distribution in phloem sap also supports this conclusion. Raf was maintained at a high level in vascular tissues, including the top part of fruit (Figure 1D and Figure 7A). Considering the high expression of *CsAGA1* in fruit top, Raf hydrolysis might occur in whole fruits including fruit top (Figure 3A and Figure 7A). Meanwhile, *CsAGA1* and *CsAGA2* were highly expressed in phloem tissues, and this indicated that RFOs were mainly hydrolysed in phloem tissues.

### 3.3. The Distinct Functions of AGAs in RFOs Hydrolysis of Cucurbitaceous Plants

In cucumber fruits, the different distribution patterns of Sta and Raf indicated the hydrolysis of Sta and Raf was distinct (Figure 1C,D) [8,9]. Gao and Schaffer identify two AGAs in melon: CmAGA1, whose enzyme activity was increased in the early stages of melon ovary, had high activity on both Raf and Sta; CmAGA2 preferred to hydrolyse Sta [18,19]. In watermelon, the RFO content is highly accumulated and monosaccharides content is decreased in *claga2* mutants [5]. These results indicated that ClAGA2 can hydrolyse both Sta and Raf in watermelon [5]. In cucumber, we found that stachyose content was significantly increased while Raf content was decreased when *CsAGA2* expression was suppressed (Figure 4H). In addition to that, the Sta and Raf content were increased when *CsAGA1* expression was suppressed (Figure 5H). Those results indicated that both CsAGA1 and CsAGA2 hydrolyse Sta, while CsAGA1 hydrolyses raffinose alone (Figure 7B). Together we conclude that AGAs have a similar but partly different function in the hydrolysis of RFOs in cucurbitaceous plants.

### 3.4. The Non-Sweet Motif within the Promoter of ClAGA2 Is Widely Distributed in Cucurbitaceous Plants

In watermelon, two single-nucleotide polymorphisms (SNPs) within the *ClAGA2* promoter regulate *ClAGA2* expression by affecting the recruitment of the transcription factor ClNF-YC2 [5]. This modification from non-sweet motif to sweet motif can effectively enhance the expression of *ClAGA2* [5], suggesting a key role of this motif in controlling *ClAGA2* expression. Furthermore, we tested whether this motif exists in the promoter of *CsAGA2*. Interestingly, the similar, non-sweet motif was also found in the promoter of *CsAGA2* (Supplemental Figure S6). To confirm whether this motif exists in other cucurbitaceous plants, we analysed the *AGA2* promoter of wax gourd, melon, and pumpkin. The non-sweet motif was also found in the promoter of wax gourd, melon but not in pumpkin (Supplemental Figure S6). So, the modification from non-sweet motif to sweet motif might enhance the *CsAGA2* expression, and this modification could be a powerful tool in genetic modification of cucurbitaceous plants.

## 4. Materials and Methods

### 4.1. Plant Growth Conditions and Materials

The cultivated lines of cucumber (*Cucumis sativus* L.) Jinchun 5 (North China ecotype) and “83-16” (North European greenhouse ecotype for fresh-eating) were obtained from Tianjin Cucumber Institute and Hezhiyuan seed industry co. LTD, respectively. A non-sweet oriental pickling melon (*Cucumis melo* L.var.conomon Thunberg., also called Vietnamese melon), was obtained from Hefeng Seed Industry Co. Ltd. (Hefei, Anhui, China) Seeds were soaked at 50 °C for 30 min and placed on moistened gauze for at 28 °C for 2 d. When the seedlings grew to the two leaves stage, the seedlings were transplanted and planted in a greenhouse at a controlled temperature (28 °C during the day, 22 °C at night) under a 16 h light photoperiod. One cucumber fruit was left in the plant.

### 4.2. Vascular Sap Sampling

The phloem sap was sampled according to Mitchell et al. [42]. For cucumber, the fruit was cut and the earliest sap on the surface was removed by clean paper to avoid contamination from broken cells. Subsequently, we collected the vascular sap in tube containing 20 μL 1% β-mercaptoethanol within a period of 2 min. One sample was collected from about 15 fruits. Five biological replicates were conducted.

### 4.3. Determination of Soluble Sugar

The extraction of phloem sap was conducted according to the method of Mitchell et al. [42]. Briefly, 20 μL phloem sap was extracted with 480 μL 80% ethanol and was dry with rotary evaporator at 40 °C. The extraction was dissolved by 500 μL ultra-pure water and the filtered by a 0.22 μm needle filter.

The extraction of soluble sugar in tissues was conducted according to the method of Hu et al. [8]. A 0.3 g sample was fully grinding and then added 10 mL 80% ethanol for 10 min. The extraction was centrifuged for 15 min at 6000× *g*. The previous steps were repeated three times and the supernatant was separated and combined. Subsequently, we placed the supernatant in a rotary evaporator at 40 °C and evaporated at reduced pressure. The deposition was dissolved in 15 mL ultra-pure water and then added 5 mL chloroform. The organic phase was removed by centrifugation and the process was repeated three times. The pH of sugar extraction was adjusted to 7.0 with 0.1 M NaOH and then evaporated on a rotary evaporator (40 °C). The deposition was dissolved by 500 μL ultrapure water and the filtered by a 0.22 μm needle filter. The pH of the aqueous phase was adjusted to 7.0 with 0.1 M NaOH and then evaporated on a rotary evaporator (40 °C). The 500 μL ultrapured water was dissolved and filtered by a 0.22 μm needle filter.

Soluble sugars were analysed with HPLC according to Miao et al. (2007) [43]. Ultrapure water acted as the mobile phase with a flow rate of 0.5 mL/min. Sample load was 5 μL. The sugar content was calculated by quantified by a refractive index detector.

### 4.4. Enzyme Activity Determination

For extracting the α-galactosidase, the plant tissue was fully grinded and soaked in an extraction buffer (50 mM HePes-NaOH, 2 mM MgCl_2_, 1 mM EDTA, 1 mM DDT) for 15 min and then centrifuged at 18,000× *g* for 30 min. The supernatant was separated and added 5% (*w/v*) PEG6000. The solution was centrifuged at 18,000× *g* for 30 min and the precipitation was dissolved with a solution buffer (25 mM HePes-NaOH, 1 mM DTT). The determination was conducted according to our previous works [25].

### 4.5. Total RNA Isolation and Expression Analysis

For vascular tissue, the vascular were separated using a capillary glass tube and subsequently immersed the separated vascular tissue in liquid nitrogen (N2). For the non-vascular tissue of stem and pedicel, the central parts were separated. For the non-vascular tissue of fruit, we separated the mesocarp. The total RNA from different cucumber tissue was extracted using RNAiso Plus (9109, TaKaRa, Dalian, China). A 1 μg sample of total RNA was used for complementary DNA (cDNA) synthesis with PrimeScript™ RT reagent Kit with gDNA Eraser (RR047Q, TaKaRa, Dalian, China). qRT-PCR was performed with One Step SYBR PrimeScript RT-PCR kit (TaKaRa, Dalian, China) on an CFX96 Touch™ Real-Time PCR Detection System (Bio-Rad, Berkeley, CA, USA) with the following cycling program: 3 min 95 °C, 40 cycles of 15 s at 95 °C, 30 s at 55 °C, and 15 s at 72 °C. The equation ratio 2^−∆∆Ct^ was applied to calculate the relative expression level. The *18S rRNA* gene (Csa2G252100) was used as an internal reference. Error bars represent the SD of three biological replicates. Primers are listed in Table S1.

### 4.6. In Situ Hybridization

To fix the fruit tissue, young cucumber fruits were soaked in FAA (70% ethanol, formaldehyde, acetic acid = 16:1:1) and then vacuumized for 10 min. The tissue slice assay was conducted following the methods as described by Yang et al. [44]. Briefly, we dehydrated the fruit tissue using graded ethanol and transparentized tissues with xylene. The fruit tissues were infiltrated and embedded using paraffin. The fruit tissues were cut into nine μm slices.

For in situ hybridization, specific fragment of *CsAGA1*, *CsAGA2* and *CsAGA3* were amplified and cloned into pGEM-TE Vector. The recombinant pGEM-TE Vector was digested using Nco I. The enzyme-digested product acted as template and the antisense probe was synthesized using Sp6 RNA polymerase. For sense probe, the recombinant pGEM-TE Vector was digested using SalI. The enzyme-digested product acted as template and the sense probe was synthesized using DIG RNA Labeling Kit (SP6/T7). The process of in situ hybridization was conducted according to a previous protocol [45]. Primers are listed in Table S1. Images were obtained using DIC microscopy (EclipseNi-U; Nikon, Tokyo, Japan). Images of slides were obtained using were observed under bright field through a Micro colour charge-coupled device (CCD) camera (Apogee Instruments, Logan, UT, USA).

### 4.7. Vector Construction

The chemical-inducible system used in this study was the CLX system, which is XVE-based (XVE for LexA-VP16-ER) [46]. The specific DNA recombination was controlled by Cre/loxP [46]. The activity of chimeric transcription factor XVE was strictly regulated by β-estrogens and XVE regulated the expression of Cre. Upon induction by β-estradiol, the sequences between two loxP sites were excised and the downstream genes were activated [46]. pX6-GFP and Psk-int vectors were obtained from Plant Molecular Biology Laboratory of Rockeffeller University [46,47]. pX6-GFP vector carries the OlexA-46 promoter. The vector contained the Cre recombinase and the expression of Cre recombinase was controlled by a transactivator XVE. Psk-int vector was used as an intermediate vector which contained multiple restriction sites on both arms of the intron [47]. For RNAi-*CsAGA1* vector, we amplified the sense fragment of *CsAGA1* and digested the sense fragment and Psk-int vector using HindIII (JH101-01, TransGen Biotech, Beijing, China). We inserted the digested sense fragment of *CsAGA1* into digested Psk-int vector using DNA Ligation Kit Ver.2.1 (6022Q, TaKaRa, Dalian, China). We amplified the anti-sense fragment of *CsAGA1* and digested the anti-sense fragment and Psk-int vector with sense fragment using BamH I and EcoR I. We inserted the digested anti-sense fragment of *CsAGA1* into digested Psk-int vector using DNA Ligation Kit Ver.2.1 (6022Q, TaKaRa, Dalian, China). We constructed RNAi-*CsAGA2* vector and RNAi-*CsAGA3* vector following the same strategy. Primers are listed in Table S1.

### 4.8. Generation of Gene Interference Lines

Through Agrobacterium (EHA105) (Agrobacterium tumefaciens)-mediated transformation, recombinant constructs were introduced into cucumber. The genetic transformation was conducted as below.

Cucumber seeds were soaked in water for 6 h and then the seed coat was removed using tweezers. To sterilize the seeds, we soaked the seeds without seed coat in 70% ethanol for 40 s and transferred the seeds in 1% sodium hypochlorite solution for 15 min. After five rinses with sterile water, the sterile seeds were transferred on MS medium (Solarbio, Beijing, China) at 28 °C–30 °C for 1 day. The 1/4 proximal, 1/4 distal parts of cotyledon were removed, and the middle part of cotyledon acted as Explants. The explants were soaked in suspension containing Agrobacterium with recombinant constructs and shook for 8 min. After co-cultivation in the dark for two days, we transferred the explants on differentiation medium (MS + 3 mg/L 6-benzylaminopurine + 0.05 mg/L 1-naphthylacetic acid + 2 mg/L abscisic acid + 2 mg/L silver nitrate + 100 mg/L kanamycin + 200 mg/L timentin). After one month, the explants were transferred on the elongation medium (MS + 1 mg/L gibberellic acid + 0.1 mg/L 6-benzylaminopurine + 0.01 mg/L 1-naphthylacetic acid + 2 mg/L silver nitrate + 100 mg/L Timentin). We cut the shoot to remove the callus and transferred the shoot to a rooting medium (MS + 0.1 mg/L 1-naphthylacetic acid + 200 mg/L Timentin).

### 4.9. β-Estradiol Treatment

To treat the fruit with β-estradiol, we added 500 µL 150 µM β-estradiol to nylon cloth and wrapped the pedicel of fruit. To avoid the photodecomposition of β-estradiol, the nylon was covered cloth with silver paper. To observe the function of CsAGAs in fruit development, the fruit was treated to the pedicel with 500 µL 150 µM β-estradiol once a day.

### 4.10. Characterization of Fruit Length, Diameter and Weight

The fruit of the 7DPA stage was selected for the characterization of fruit length, diameter, and weight. For fruit diameter, the length of the thickest part was measured. For fruit length, the length from the top part of the fruit to the pedicel was measured. For fruit dry weight, the fruit was cut into pieces, and we remove moisture until the weight was constant at 60 °C. Fifteen biological replicates were conducted.

### 4.11. Accession Numbers

Sequence data from this article can be found in the CuGenDB (http://cucurbitgenomics.org/, accessed on 2 December 2021) under the following accession nos.: CsGAL1, Csa5G580620; CsGAL2, Csa5G580630; CsGAL3, Csa5G220910; CsAGA1, Csa4G631570; CsAGA2, Csa4G167980; CsAGA3, Csa1G229500; 18S rRNA, Csa2G252100.

## 5. Conclusions

In this study, our results showed that RFOs were metabolized in both pedicel and fruit. In addition, *CsAGAs* but not *CsGALs* were highly expressed in vascular tissues by qRT-PCR and in situ hybridization. Compared with GALs, AGAs showed higher activity in RFO hydrolysis. By inducible silencing the CsAGAs, the growth of cucumber fruits and RFO catabolism were inhibited in both *CsAGA1*-RNAi and *CsAGA2*-RNAi plants, but not in *CsAGA3*-RNAi plants. Further investigation suggests that *CsAGA2* is mainly responsible for Sta catabolism, while CsAGA1 catalyses both Sta and Raf metabolism. Our results provide important information for uncovering the enigma of RFOs unloading in cucumber fruits and potential tools to improve cucumber yield and quality.

## Data Availability

The sequences used in this study are available online at http://cucurbitgenomics.org/, accessed on 2 December 2021.

## Supplementary Materials

The following are available online at https://www.mdpi.com/article/10.3390/ijms222413285/s1.
