# Supplementary material for: CsAGA1 and CsAGA2 Mediate RFO Hydrolysis in Partially Distinct Manner in Cucumber Fruits
Source: Int J Mol Sci. 2021 Dec 10;22(24):13285. doi: 10.3390/ijms222413285 (PMC8706097; doi:10.3390/ijms222413285)
Supplement: Supplementary file 1 [file ijms-22-13285-s001.zip › ijms-1489613-supplementary.pdf]

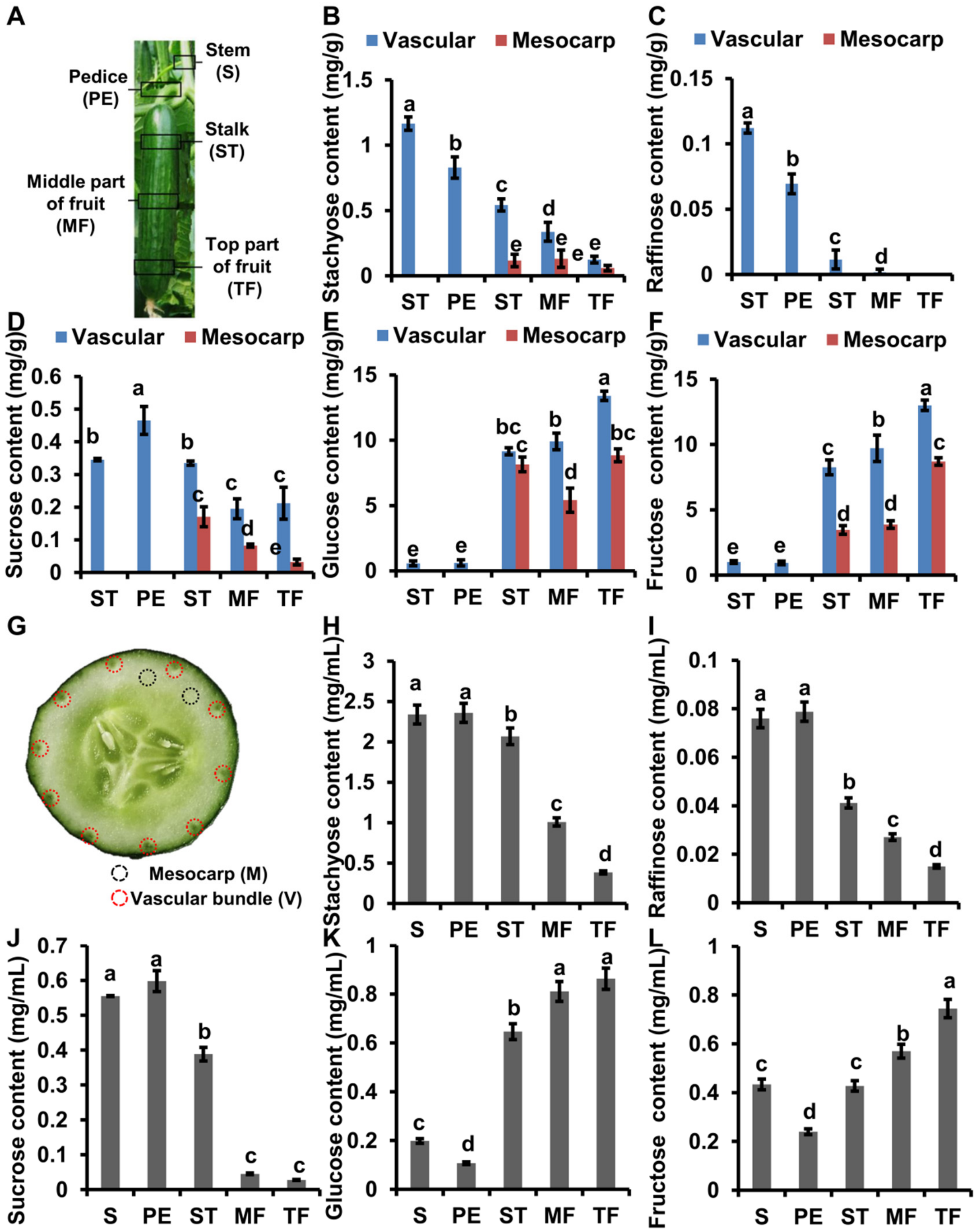

Supplemental Figure S1. The distribution of soluble sugar contents in "83-16".

(A) The schematic diagram for location of the sampling in fruit. Stem (S), pedicel (PE), vascular tissue and non-vascular tissue of stalk (ST), basal part (BF), middle part (MF) and top part (TF) of fruit were used for the analysis of soluble sugar content. (B-F) The Sta (B), Raf (C), Suc (D), Glu (E) and Fru (F) content in different tissue of fruit were analyzed by HPLC. (G) The schematic diagram shows mesocarp (M) and

vascular bundle (V). (H-L) The Sta (H), Raf (I), Suc (G), Glu (K) and Fru (L) contents in phloem sap of fruit were analyzed by HPLC. The phloem sap from pedicel (PE), stalk (ST), basal part (BF), middle part (MF) and top part (TF) of fruit were collected for measurement.

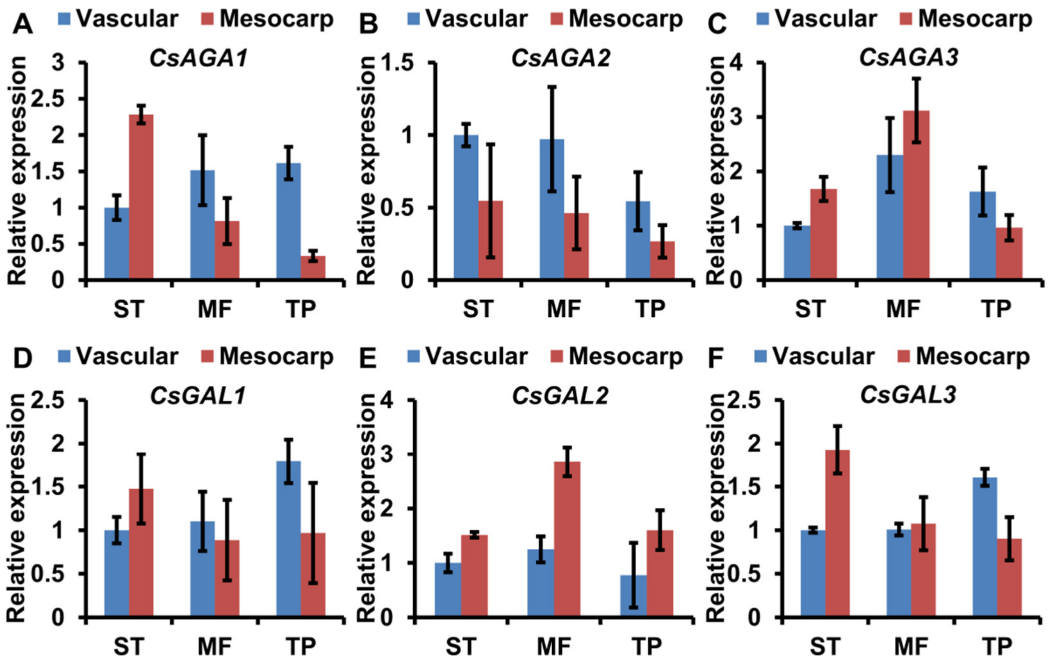

Supplemental Figure S2. The expression of *α-galactosidase* genes in vascular and mesocarp in different parts of "83-16" fruit.

(A-C) Relative expression show that alkaline forms *α-galactosidase* genes (*CsAGA1* (A), *CsAGA2* (B) and *CsAGA1* (C)) were preferred expressed in vascular tissue. (D-E) Relative expression showed that acid forms *α-galactosidase* genes (*CsGAL1* (D), *CsGAL2* (E) and *CsGAL3* (F)) did not show the prefer expression in vascular tissue. The bars represent standard deviation (SD) of three biological replicates. *18S rRNA* acted as reference gene and relative amounts were normalized with respect to the expression in vascular tissue.

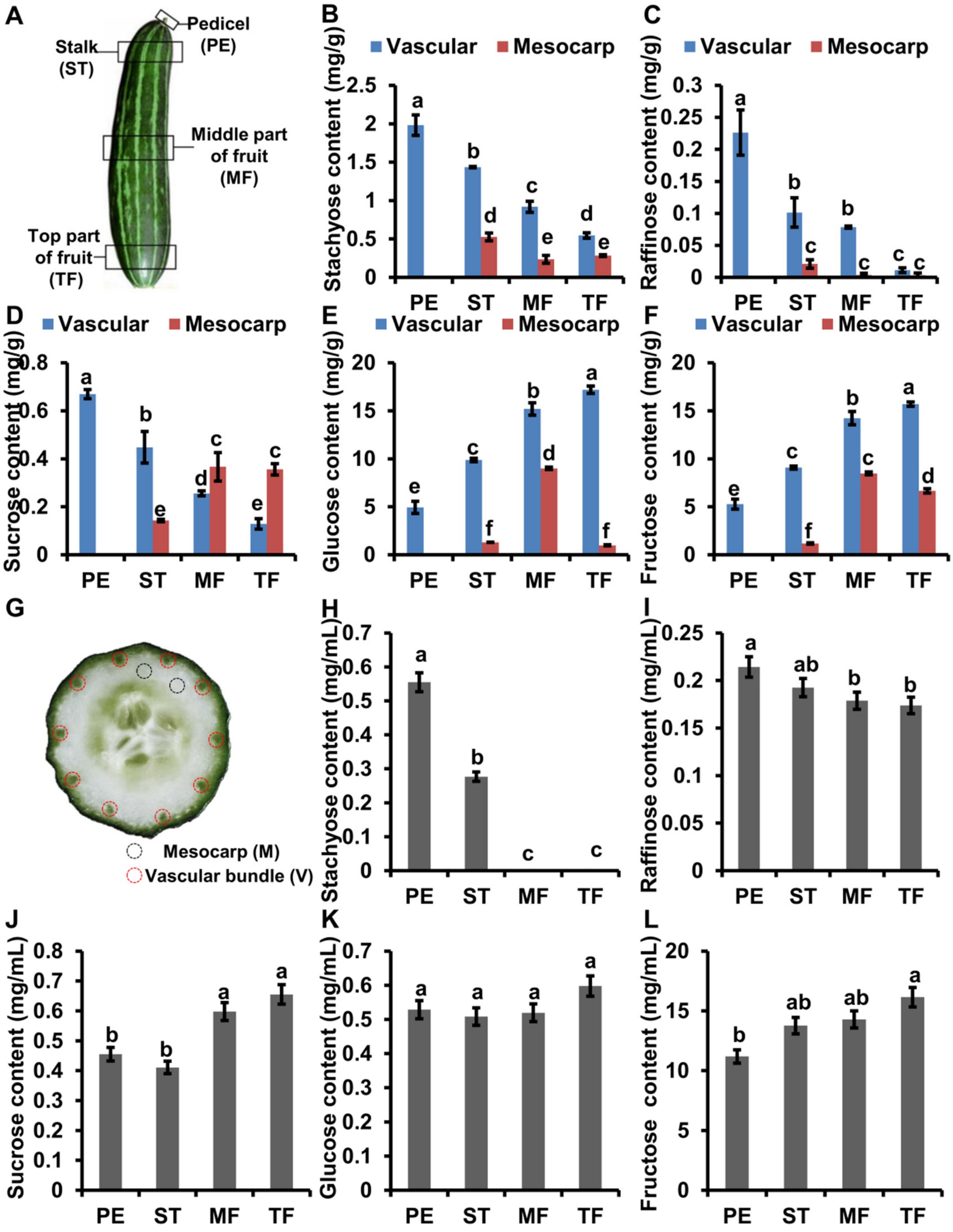

Supplemental Figure S3. The distribution of soluble sugar in melon.

(A) The schematic diagram for location of the collection sample in fruit. Pedicel (PE), Vascular tissue and non-vascular tissue of stalk (ST), middle part (MF) and top part (TF) of fruit were used for the analysis of soluble sugar content. (B-F) The Sta (B), Raf (C), Suc (D), Glu (E) and Fru (F) content in different tissues of fruit were analyzed by HPLC. (G) The schematic diagram shows mesocarp (M) and vascular

bundle (V). (H-L) The Sta (H), Raf (I), Suc (G), Glu (K) and Fru (L) content in phloem sap of fruit were analyzed by HPLC. The phloem sap from pedicel (PE), stalk (ST), middle part (MF) and top part (TF) of fruit were collected for analysis.

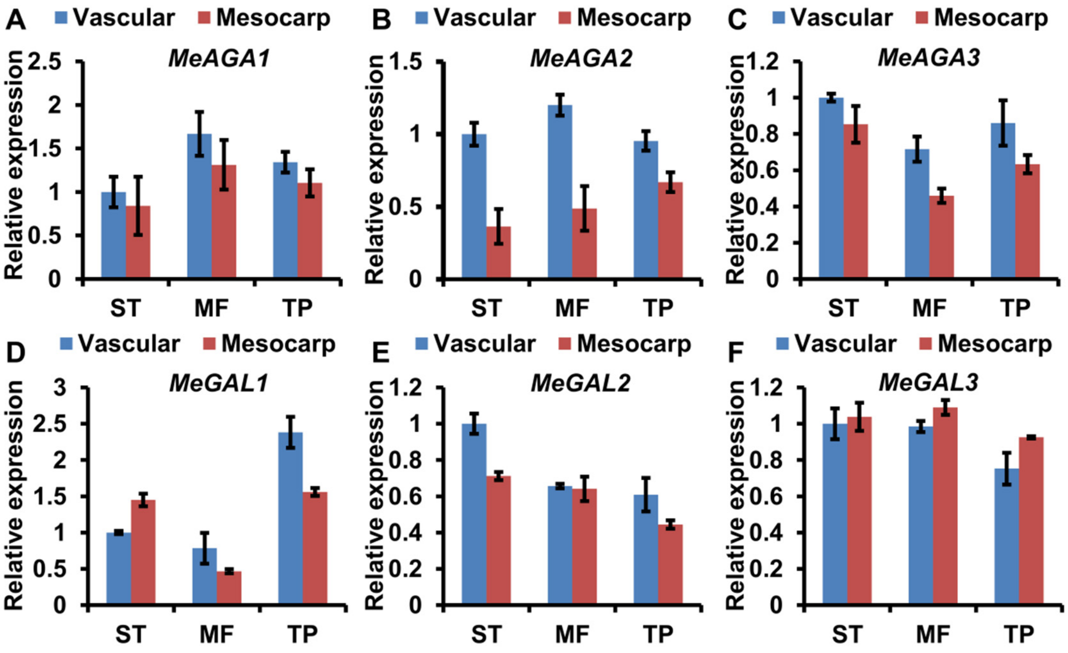

Supplemental Figure S4. The expression of *α-galactosidase* genes in vascular and mesocarp in different part of melon fruit.

(A-C) Relative expression show that *α-galactosidase* genes of alkaline forms (*MeAGA1* (A), *MeAGA2* (B) and *MeAGA1* (C)) were prefer expressed in phloem. (D-E) Relative expression show that *α-galactosidase* genes of acid forms (*MeGAL1* (D), *MeGAL2* (E) and *MeGAL3* (F)) were not preferred expressed in vascular tissue. The bars represent standard deviation (SD) of three biological replicates. *18S rRNA* acted as reference gene and relative amounts were normalized with respect to the expression in vascular tissue.

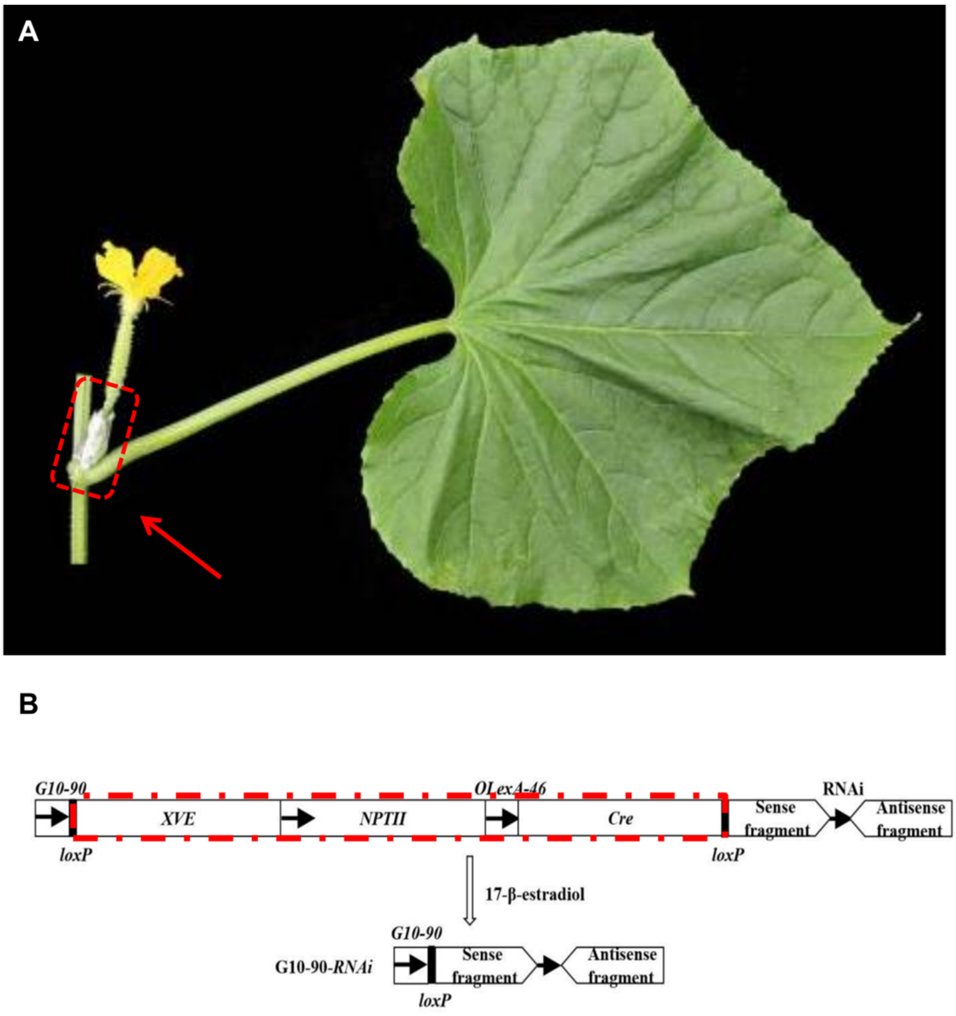

Supplemental Figure S5. β-estradiol treatment using wrapping method.

(A) Nylon cloth containing 500μL β-estradiol wrapped the pedicel. For qRT-PCR, we sampled the target tissue after β-estradiol treatment. For observe the regulation of CsAGAs on fruit development, we treat fruit pedicel with β-estradiol once a day. (B) The diagram of β-estradiol induced silencing expression vector. After β-estradiol treatment, the elements between *loxP* are deleted and the subsequent sequences are induced expression.

Water melon-sweet: **CTTGG**GTTGGT**GTTGGTG**

Water melon-non-sweet: **CTTAG**GTTGGT**GTTAGTG**

Cucumber: -185 **CTTAG**GTTGAT**GTTAGTG**-168

Melon: -189 **CTTAG**GTTGAT**GTTAGTG**-172

Wax melon: -186 **CTTAG**GTTGGT**GTTAGTG**-169

Supplemental Figure S6. The motif comparison of *AGA2* promoter.

The numbers show the motif location of *AGA*2 promoter. Bold letters show the binding sites of NF-Y2.

Supplemental Table S1. The list of primers used for this pape.

Primer	Sequence(5'-3')	Purpose
Q-CsAGA1-FW	GAGAATCCCGACACAATCACAGG	
Q-CsAGA1-RV	TCGATGCATCTTGTGGTAGGTAAAC	
Q-CsAGA2-FW	TGCAAGTAGGCTGTCTGGAATAAAAG	
Q-CsAGA2-RV	CCACCCCAATAACCAGCCAAAG	
Q-CsAGA3-FW	CCGCAATCAGAGAGAATTCGAAGTT	
Q-CsAGA3-RV	GATGATCCGTATTCCTCCATGTCCT	
Q-CsGAL1-FW	TTACGAACACAGAAGTCATTGCAGT	
Q-CsGAL1-RV	GTCCCATTGTGCCGAGATTGC	qRT-PCR
Q-CsGAL2-FW	CTGAGTTTGATTCTGCTTCCTCTAGG	
Q-CsGAL2-RV	CTGATATCCTAATGCAGCAAGTCCG	
Q-CsGAL3-FW	TGATACCTCCAAGTACGGCATAC	
Q-CsGAL3-RV	CGTACACATAACCTAACTCAGCC	
Q-18S-FW	GCTGATTGCTGATTGGATGTGACAT	
Q-18S-RV	TCCATAGAACCACAGCGACTCTTT	
in situ-CsAGA1-FW	TCCTATAGGTTTGATGAAG	
in situ-CsAGA1-RV	GTTCTCATAGTTCAATTC	
in situ-CsAGA2-FW	TCTCCCTGATGATCGACCTC	
in situ-CsAGA2-RV	GTTCCATCTA TACATCTCCT	In situ hybridization
in situ-CsAGA3-FW	AGGAGGAAGGAGATGAGAA	
in situ-CsAGA3-RV	GGAATACGGATCATCAAGC	
AGA1-F-FW	ATGGTCGACAGTTCAAACGTG	
AGA1-F-RV	CTCAAGCTTTCCCATCCATCC	
AGA1-R-FW	CGAGGATCCAGTTCAAACGTGTC	
AGA1-R-RV	CGTGAATTCTCCCATCCATCC	
AGA2-F-FW	GACGTCGACCGGCCACTATCACCAT	
AGA2-F-RV	GCCAAGCTTACATTAATGACAGAT	Transgenosis
AGA2-R-FW	GACGGATCCCACTATCACCAT	
AGA2-R-RV	CGCGAATTCACATTAATGACAG	
AGA3-F-FW	TTC GTCGAC TACTCGTCGGCGAAAC	
AGA3-F-RV	GAG AAGCTT CCTATAATCGGCACCT	
AGA3-R-FW	TTC GGATCC TACTCGTCGGCGAAAC	
AGA3-R-RV	GAG GAATTC CCTATAATCGGCACCT	
